# Dietary Intake of Parkinson's Disease Patients

**DOI:** 10.3389/fnut.2020.00105

**Published:** 2020-07-21

**Authors:** Florence Baert, Christophe Matthys, Randy Mellaerts, Dirk Lemaître, Geertrui Vlaemynck, Veerle Foulon

**Affiliations:** ^1^Department Technology and Food, Flanders Research Institute for Agriculture, Fisheries and Food, Melle, Belgium; ^2^Clinical and Experimental Endocrinology, Department of Chronic Diseases, Metabolism and Ageing, University of Leuven, Leuven, Belgium; ^3^Department of Endocrinology, University Hospitals Leuven, Leuven, Belgium; ^4^Clinical Pharmacology and Pharmacotherapy, KU Leuven, Leuven, Belgium; ^5^Parki's KookAtelier, Leuven, Belgium; ^6^Nutrition and Dietetics, UC Leuven-Limburg, Leuven, Belgium

**Keywords:** Parkinson disease, dietary intake, fiber, meal pattern, levodopa

## Abstract

**Background and Aims:** Dietary management, as an adjuvant therapy in Parkinson's disease (PD), provides clear benefits to patients. However, baseline information about the usual dietary intake of Parkinson's patients is lacking.

**Methods:** We conducted an observational cross-sectional study, investigating the dietary intake in Belgian PD patients, as well as their medication use and knowledge of possible food-drug interactions. A dietary record of 2 non-consecutive days, allowing the calculation of usual intake, was used. Medication use and knowledge of food-drug interactions were investigated using a self-administered questionnaire.

**Results:** The nutrient (both macro and micro) intake in this study was similar to the dietary pattern of the general Belgian population. However, results showed that the PD population had a high dietary fiber intake of 26.2 ± 7.7 g/day, which is in line with the recommended intake. The majority of the PD patients had an inadequate intake of vitamin D and iron (respectively, 55.9 and 76.5% of all participants). When looking into the knowledge about food-drug interactions, the majority of the PD patients claimed to be aware of the food-drug interaction between dietary proteins and levodopa. However, only 18.2% of the patients took all doses of levodopa out of meals.

**Conclusion:** Our results show that monitoring of dietary intake in PD patients is of importance to detect possible micronutrient insufficiencies. Patients should receive professional guidance in optimizing their diet to accommodate for different complaints inherent to PD, including constipation. Furthermore, the knowledge of patients regarding the importance of correct medication intake should be improved.

## Introduction

Parkinson's disease (PD) is the second most prevalent neurodegenerative disorder, characterized by dopaminergic neuronal cell loss and the presence of aggregated α-synuclein protein, the so-called Lewy bodies, in the central nervous system (CNS) (1). Although PD is mainly recognized as a movements disorder, the disorder is defined by both motor and non-motor symptoms. PD patients have an increased risk of malnutrition, however the underlying mechanisms are not fully elucidated (2). Both weight loss and weight gain have been reported in PD. Even when body weight is stable, there seems to be a redistribution in body composition from muscle to fat, suggesting PD patients are at risk of sarcopenia (3). Symptoms such as chewing- and swallowing difficulties, loss of smell and constipation may even further increase the risk of malnutrition in PD (3, 4).

In 2011, the British Dietetics Association (BDA) produced, in partnership with Parkinson's UK, a best practice guideline for dietitians on the management of Parkinson's disease (PD), emphasizing the importance of nutritional management in different stages of the neurodegenerative disorder (4). Although no specific diet is required, different symptoms or consequences of PD should be taken into consideration (4). It concerns the increased risk of malnutrition, various gastro-intestinal and sensory deficits and food-drug interactions. Nutrient composition, but also timing of consumption of meals plays a major role in the PD management (3–6).

Constipation is a highly prevalent symptom inherent to PD, which often occurs before the onset of motor symptoms and negatively impacts the quality of life (5). Hardly any evidence is available about the effect of fiber consumption on constipation in PD, although fiber improves stool frequency and consistency in adults with chronic idiopathic constipation (5, 6).

The timing and amount of dietary protein intake is of importance in PD, as amino acids and levodopa (the most frequently used drug in PD) are absorbed via the large-neutral amino acid transporter, both at the level of the small intestine and of the blood-brain barrier. To avoid competition and the resulting lower bioavailability of levodopa, it is recommended to take the medication 30 min prior or 1 h after the meal (7).

Some studies suggest a protective effect of intake of different vitamins and antioxidants in PD, but further investigation is warranted (8). A ketogenic diet has also been proposed as treatment of motor dysfunction in neurologic disease, but lack of clinical data and risk of adverse events currently prevents the therapeutic use (9).

In spite of the importance of nutritional management in PD, the information about dietary habits of PD patients is scarce. Dietary studies may grant an interesting insight into the diet of PD patients and could provide on how to reduce different symptoms and thus improve patients' quality of life.

Therefore, the objective of this study is to describe the nutrient intake of Belgian PD patients, compare these intakes to the general nutrient recommendations, and to investigate their medication taking behavior and knowledge of potential food-drug interactions.

## Methods

An observational, cross-sectional study was conducted between November and December 2015 in PD patients. The study consisted of a general questionnaire (focusing on medication taking behavior and knowledge about drug-food interactions) and a dietary record of 2 non-consecutive days. The study protocol complied with the Helsinki declaration and was approved the 26th of October 2015 by the Ethics Committee of the University of Leuven (Reference MP05560-S58394).

A convenience sample of PD patients was recruited through their participation in cooking workshops organized by patient's organization “Parki's KookAtelier” (Meersman)^1^ Inclusion criteria were self-reported diagnosis of PD and self-reported intake of any type of PD medication, cross-checked by the research team and the patient's physician.

The dietary record protocol was based on Gesquiere et al. and adapted to the patient population (10). The record needed to be completed during 2 non-consecutive days in the week before the workshop and included dietary and timing of medication intake. Dietary recording during 1 day was defined as everything consumed by the participant during 24 h (from midnight to midnight). A specific time structure was used: consumption in the morning, before noon, at noon, in the afternoon, in the evening and in the late evening. The participant was asked to indicate the timing of every consumption, as well as the timing of every medication intake. A dietary record of 2 non-consecutive days was chosen, since 2 non-consecutive days are the minimum number of days needed to properly estimate an individual's intake (11). Non-consecutive days were chosen, since foods eaten on consecutive days may be related (11). Furthermore, longer recording periods often induce reporting fatigue, furthermore participants develop the practice of filling out the dietary record retrospectively (12). Although longer recording periods are recommended in studies with a small sample size, a dietary record of 2 non-consecutive days remained our choice to avoid reporting fatigue and participant dropout (12). Especially, since PD patients often already suffer from both physical and mental fatigue (13).

The general questionnaire was completed during the workshop and consisted of multiple choice questions about socio-demographic characteristics, medication use, changes in the diet and their underlying reason, knowledge about food-drug interactions, and the sources of information concerning food-drug interactions.

After completion, the dietary records were processed and coded by dietitians. For determination of nutrient intake, the Belgian Food Composition DataBase (FCDB, NUBEL) was used (14). Based on the actual intake, the usual dietary intake was calculated using the Multiple Source Method (MSM). MSM is a statistical analysis that allows the estimation of usual dietary intake based on two or more short-term measurements (15). Afterwards, the percentage of macronutrients of the daily total energy intake was calculated, using the Atwater factors. All dietary intake data are presented as usual intake. The data were compared with the general nutrient recommendations (16, 17), since PD-specific recommendations are lacking. The general nutrient recommendations of the Institute of Medicine (IOM) were used, since the IOM guidelines are more comprehensive regarding the Estimated Average Requirements (EAR) for micronutrients. The energy intake of the participants was compared with the average requirements (AR) of men and women (aged 60–70) based on a range of energy intakes using the physical activity level (PAL) 1.4–1.8. There is no consensus on the PAL in PD, therefore a range was used. Micronutrient intake was compared with the EAR (age categories 19–70+), to determine the prevalence of inadequate intake using the EAR Cut-off method (16). When the EAR differed between men and women, both values were taken into account.

As for the data from the general questionnaire, the percentage of correct intake moments of levodopa per patient was calculated. The evaluation of the intake moment (correct = 30 min prior or 1 h after consumption of a meal) was based on the reported intake of medication in the dietary records. PD patients who did not administer levodopa or did not register a time point of medication intake were excluded from this analysis.

The normality of data was assessed using the Shapiro-Wilk test. Possible associations between categorical data of different socio-demographic characteristics were analyzed using Pearson's Chi Square test. A student's *t*-test was used to analyze the difference in nutrient intake according to gender. A Mann-Whitney *U*-test was used if the data was not normally distributed. The level of statistical significance was determined as *p* < 0.05. The data were analyzed with Statistica 13.1.

## Results

**Figure 1:**
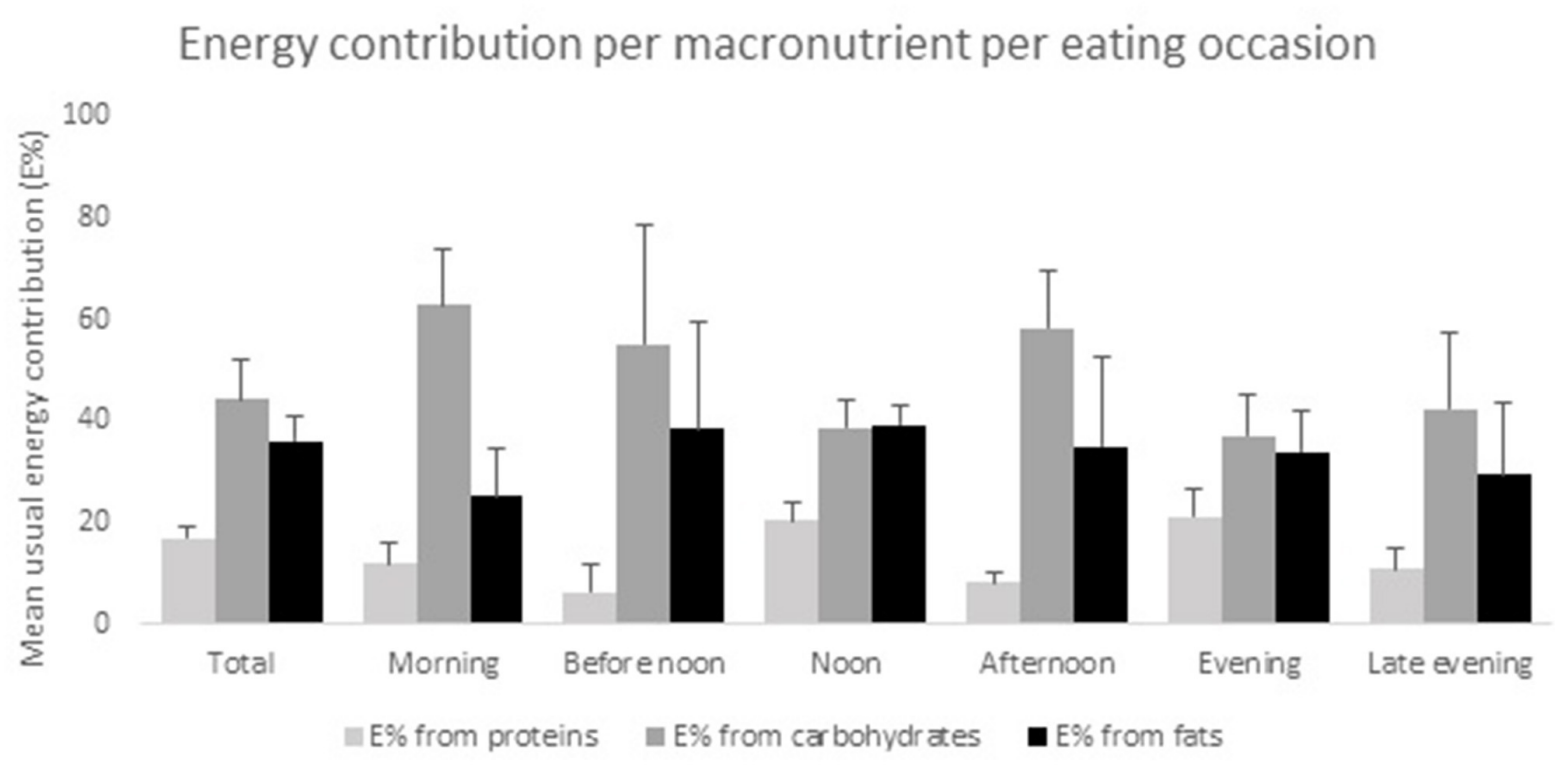
Energy contribution per macronutrient per eating occasion. All results are presented as mean E% ± SD, *n* = 34.

In total, 52 men and 22 women aged 49–84 years were included in the study. Four patients were excluded from the study due to the lack of information about PD medication intake. Of the 41 food records that were received, seven diaries were excluded, either because of incompleteness or because of non-compliance to the instructions to complete the record during 2 non-consecutive days. A detailed overview of the demographics of the study population is shown in Table 1. The mean age at which participants received their diagnosis of PD was 59, while the diagnosis was predominantly made by a neurologist, namely in 83.8% of all participants. The majority, 47.8%, of the participants described their financial situation as average,. The main types of education the participants received were secondary and polytechnic education, respectively, by 31.1 and 39.2%. The majority of the participants received practical (67.6%) and/or emotional (51.4%) support from their environment. Most participants, 86.5%, lived in a multi-person household. The mean daily energy intake was 2,194 ± 581 kcal, with lunch (670 ± 124 kcal) and dinner (632 ± 179 kcal) supplying the most energy. Figure 1 provides an overview of the energy contribution per macronutrient for each eating occasion. Table 2 shows the results of mean energy, macro- and micronutrient intake in men and women, compared with the nutrient recommendations.

**Table 1:** Socio-demographic characteristics of study population.

	**Men (*n* = 52)**	**Women (*n* = 22)**	***p*-value**	**Total (*n* = 74)**
**Age**
Age (years), mean (minimum–maximum)	67 (49–84)	69 (54–80)	1.00	67 (49–84)
Age at time of diagnosis (years), mean (minimum–maximum)	58 (35–82)	60 (38–80)	0.35	59 (35–82)
**Diagnosis made by**			0.20	
General practitioner, *n* (%)	9 (17.3)	1 (4.5)		10 (13.5)
Neurologist, *n* (%)	42 (80.8)	20 (90.9)		62 (83.8)
Psychiatrist, *n* (%)	0 (0.0)	1 (4.5)		1 (1.4)
Neurosurgeon, *n* (%)	1 (1.9)	0 (0.0)		1 (1.4)
**Financial situation**			0.77	
Easy, *n* (%)	9 (17.3)	3 (14.3)		12 (16.4)
Mediocre, *n* (%)	13 (25.0)	4 (19.0)		17 (23.3)
Average, *n* (%)	24 (46.2)	11 (52.4)		35 (47.8)
Difficult, *n* (%)	4 (7.7)	3 (14.3)		7 (9.6)
Very difficult, *n* (%)	2 (3.8)	0 (0.0)		2 (2.7)
**Highest obtained degree**			0.01	
University, *n* (%)	9 (17.3)	1 (4.5)		10 (13.5)
Polytechnic, *n* (%)	25 (48.1)	4 (18.2)		29 (39.2)
Secondary education, *n* (%)	11 (21.2)	12 (54.5)		23 (31.1)
Primary education, *n* (%)	5 (9.6)	5 (22.7)		10 (13.5)
None, *n* (%)	2 (3.8)	0 (0.0)		2 (2.7)
**Type of social support**			0.62	
Financial, *n* (%)	3 (5.8)	2 (9.1)		5 (6.8)
Practical, *n* (%)	34 (65.4)	16 (72.7)		50 (67.6)
Emotional, *n* (%)	24 (46.2)	14 (63.6)		38 (51.4)
None, *n* (%)	11 (21.2)	1 (4.5)		12 (16.2)
**Household composition**			0.03	
Multi person household	48 (92.3)	16 (72.7)		64 (86.5)
Living alone	4 (7.7)	6 (27.3)		10 (13.5)

**Table 2:** Mean energy intake and mean intakes of macro- and micronutrients by sex group.

	**Total (*n* = 34)**	**Men (*n* = 24)**	**Women (*n* = 10)**	***P*-value**	**Dietary recommendations (DR)**	
					**Men**	**Women**
Energy intake (kcal), mean (SD), % achieving DR	2,194 (±581), 61.8	2,230 (±632), 62.5	2,109 (±455), 60	0.8	1,744–3,025	1,283–2,173
**Macronutrients**						
Water (l), mean (SD), % achieving DR	2.1 (±0.5), 2.9	2.1 (±0.5), 0	2.1 (±0.5), 10	0.9	3.7 l	2.7 l
Protein (E%), mean (SD), % achieving DR	16.8 (±4.0), 100	17.1 (±2.4), 100	17.0 (±2.2), 100	0.8	10–35 E%	
Carbohydrates, total (E%), mean (SD), % achieving DR	44.3 (±14.2), 56	44.8 (±7.4), 58.3	43.3 (±8.3), 50	0.8	45–65 E%	
Mono- and disaccharides (E%), mean (SD)	20.4 (±6.1)	20.8 (±5.6)	19.5 (±4.3)	0.4	NA	
Starch (E%), mean (SD)	20.8 (±6.1)	21.4 (±6.0)	19.2 (±6.5)	0.4	NA	
Dietary fiber (g), mean (SD), % achieving DR	26.2 (±7.7), 50	26.2 (±8.5), 37.5	26.1 (±8.5), 80	0.2	30 g	21 g
Fat, total (E%), mean (SD), % achieving DR	35.9 (±10.7), 50	35.1 (±4.9), 58.3	37.8 (±5.7), 30	0.5	20–35 E%	
SFA (E%), mean (SD)	14.3 (±3.1)	14.2 (±3.0)	14.5 (±3.5)	0.7	NA	
MUFA (E%), mean (SD)	12.1 (±2.1)	12.1 (±2.1)	12.0 (±1.9)	0.6	NA	
PUFA (E%), mean (SD), % achieving DR	6.7 (±1.4), 88.2	6.4 (±1.0), 91.7	7.5 (±1.9), 80	0.7	5–10 E%	
Cholesterol (mg), mean (SD)	291.8 (±116.4)	292.6 (±107.1)	289.8 (±142.5)	0.8	NA	
**Micronutrients**						
Vitamin A (μg), mean (SD), % achieving DR	1123.5 (±146.7), 100	1089.1 (±138.6), 100	1206.2 (±138.1), 100	0.03	625	500
Vitamin B1 (mg), mean (SD), % achieving DR	1.8 (±0.7), 88.2	1.9 (±0.7), 87.5	1.5 (±0.5), 90	0.2	1.0	0.9
Vitamin B2 (mg), mean (SD), % achieving DR	1.6 (±0.3), 100	1.6 (±0.3), 100	1.6 (±0.3), 100	0.9	1.1	0.9
Vitamin B12 (μg), mean (SD), % achieving DR	7.2 (±0.3), 100	7.2 (±0.3), 100	7.1 (±0.4), 100	0.5	2.0	2.0
Vitamin C (mg), mean (SD), % achieving DR	153.7 (±26.8), 100	150.5 (±23.8), 100	161.5 (±33.1), 100	0.2	75	60
Vitamin D (μg), mean (SD), % achieving DR	10.1 (±3.9), 44.1	10.0 (±4.3), 37.5	10.6 (±2.9), 60	0.7	10	10
Iron (mg), mean (SD), % achieving DR	4.4 (±3.0), 23.5	4.0 (±2.7), 16.7	5.2 (±3.5), 40	0.4	6.0	5.0
Copper (μg), mean (SD), % achieving DR	2053.4 (±200.4), 100	2105.5 (±200.3), 100	1928.2 (±141.5), 100	0.02	700	700
Zinc (mg), mean (SD), % achieving DR	12.7 (±4.6), 85.3	13.0 (±4.9), 79.2	11.7 (±3.7), 100	0.4	9.4	6.8

*The reference for all dietary recommendations is the Institute of Medicine. The AR of energy intake for both men (length 170–185 cm, aged 60–70) and women (length 145–160 cm, aged 60–70) is a range of energy intakes based on PAL 1.4–1.8. RI for protein, total carbohydrates and fat; AI for fatty acids and dietary fiber are presented. AR, average requirement; EAR, estimated average requirement; RI, reference intake; AI, adequate intake; SFA, saturated fatty acids; MUFA, mono-unsaturated fatty acids; PUFA, poly-unsaturated fatty acids; SD, standard deviation; NA, not applicable; DR, Dietary recommendations. Significance at 0.05 level*.

None of the participants had a protein-derived energy contribution lower than 10 E%, or above 35 E%. The largest E-intake of proteins was during lunch time (20.4 ± 3.6 E%) and during dinner (21.2 ± 5.2 E%) (Figure 1). Our results show that 44% of the participants (50.0% of women and 41.7% of men), had a total carbohydrate intake lower than 45 E%. During breakfast, morning and afternoon snack, the energy contribution of carbohydrates was 62.9 ± 10.9, 55.1 ± 23.6, and 58.3 ± 11.5 E%, respectively (Figure 1). In 50% of the participants (respectively, 20% women and 62.5% men), a dietary fiber intake below the recommendations was found, resulting in a daily intake ranging from 12.0 to 23.5 g. Mean daily fiber intake was 26.2 ± 7.7 g/day, where at noon (8.8 ± 2.9 g) and in the evening (8.4 ± 4.9 g) the highest amount of dietary fiber was consumed. Overall, 70% of the female and 41.7% of the male participants had a total fat intake above 35 E%. Mean SFA intake of all participants was 14.3 ± 3.1 E%. A PUFA intake below 5 E% was only observed in 8.3% of the male participants, while 20% of the female and none of the male participants had a PUFA intake higher than 10 E%.

None of the participants had a vitamin A intake below the EAR. Our results showed that 11.8% of the patients had an inadequate intake of vitamin B1 (thiamin) (respectively, 10% women and 12.5% men). Results of vitamin D intake showed that 62.5% of men and 40% of women had a mean daily intake below 10 μg. No other insufficiencies of vitamin intake were found.

The results for iron intake showed that 83.3% of men and 60% of women had an intake below the EAR. For zinc intake, we found that 20.8% of men but none of the women had an insufficient intake.

Of all participants, 55.4% noted a change in their dietary habits since being diagnosed with PD. However, 89.0% of all patients never consulted a disease-specific dietician. The majority of the participants changed the composition of their diet, namely 48.7%, see Table 3 The main reasons for changing dietary habits were constipation (52.6% of the participants), chewing and swallowing difficulties (42.1% of the participants) and loss of smell (34.2% of the participants).

**Table 3:** Overview of participant's changes in dietary habits, the reasons behind it, and potential problems during meal preparation.

	**Total**
**Dietary habits (*****n*** **=** **74)**	
Made a change in their dietary habits, *n* (%)	41 (55.4)
**Dietary consult regarding PD (*****n*** **=** **73)**	
Consulted a dietitian once, *n* (%)	2 (2.7)
Consulted a dietitian multiple times, *n* (%)	3 (4.1)
Is currently consulting a dietitian, *n* (%)	3 (4.1)
Never consulted a dietitian, *n* (%)	65 (89.0)
**How did dietary habits change (*****n*** **=** **39)**	
Composition of diet, *n* (%)	19 (48.7)
Food structure of diet, *n* (%)	8 (20.5)
Dietary pattern, *n* (%)	9 (23.1)
Use of supplements, *n* (%)	11 (28.2)
Other, *n* (%)	6 (15.4)
**Reasons for changing dietary habits (*****n*** **=** **38)**	
Chewing and swallowing problems, *n* (%)	16 (42.1)
Constipation, *n* (%)	20 (52.6)
Reduced sense of taste, *n* (%)	9 (23.7)
Reduced sense of smell, *n* (%)	13 (34.2)
Reduced ability to cook, *n* (%)	8 (21.1)
Drug side effects, *n* (%)	6 (15.8)
Was advised to by someone, *n* (%)	5 (13.2)
Other, *n* (%)	8 (21.1)
**Person responsible for meal preparatio*****n*** **(*****n*** **=** **73)**	
Participant, *n* (%)	29 (39.7)
Partner, *n* (%)	46 (63.0)
Children, *n* (%)	1 (1.4)
Grandchildren, *n* (%)	1 (1.4)
Home care service, *n* (%)	4 (5.5)
Other, *n* (%)	5 (6.9)
**Problems during cooking (*****n*** **=** **28)**	
Experiences problems during cooking, *n* (%)	17 (60.7)
Problems during cooking due to resting tremor, *n* (%)	1 (5.9)
Problems during cooking due to rigidity, *n* (%)	2 (11.8)
Problems during cooking due to inertia, *n* (%)	7 (41.2)
Other, *n* (%)	5 (29.4)

Of all participants, 39.7% were responsible for their own meal preparation while 63.0% of the participants were supported by their partner. Of the participants who cooked for themselves, 60.7% experienced problems during cooking: the majority, 41.2%, suffered from inertia. Data is shown in Table 3.

Of all participants, 97.2% reported levodopa use, of whom 64.4% were aware of the food-drug interaction between dietary proteins and levodopa. Only 6.8% of the participants remembered having been informed about this interaction by their pharmacist. Data is shown in Table 4.

**Table 4:** Overview of medication intake and participant's knowledge and information source of food-drug interactions.

	**Total**
**Medication intake (*****n*** **=** **71)**	
Levodopa, *n* (%)	69 (97.2)
Anticholinergics, *n* (%)	2 (2.8)
Dopamine agonists, *n* (%)	45 (63.4)
Monoamino-oxidase B inhibitor, *n* (%)	41 (57.7)
Amantadine, n (%)	3 (4.2)
**Knowledge of food-drug interactions (*****n*** **=** **73)**	
Aware of interaction between dietary proteins and levodopa, *n* (%)	47 (64.4)
**Information source of food-drug interactions (*****n*** **=** **74)**	
General practitioner, *n* (%)	12 (16.2)
Neurologist, *n* (%)	19 (25.7)
Pharmacist, *n* (%)	5 (6.8)
Dietitian, *n* (%)	1 (1.4)
Home care service, *n* (%)	1 (1.4)
The internet, *n* (%)	8 (10.8)
Patient's organizations, *n* (%)	16 (21.6)
Other	9 (12.2)

**Figure 2:**
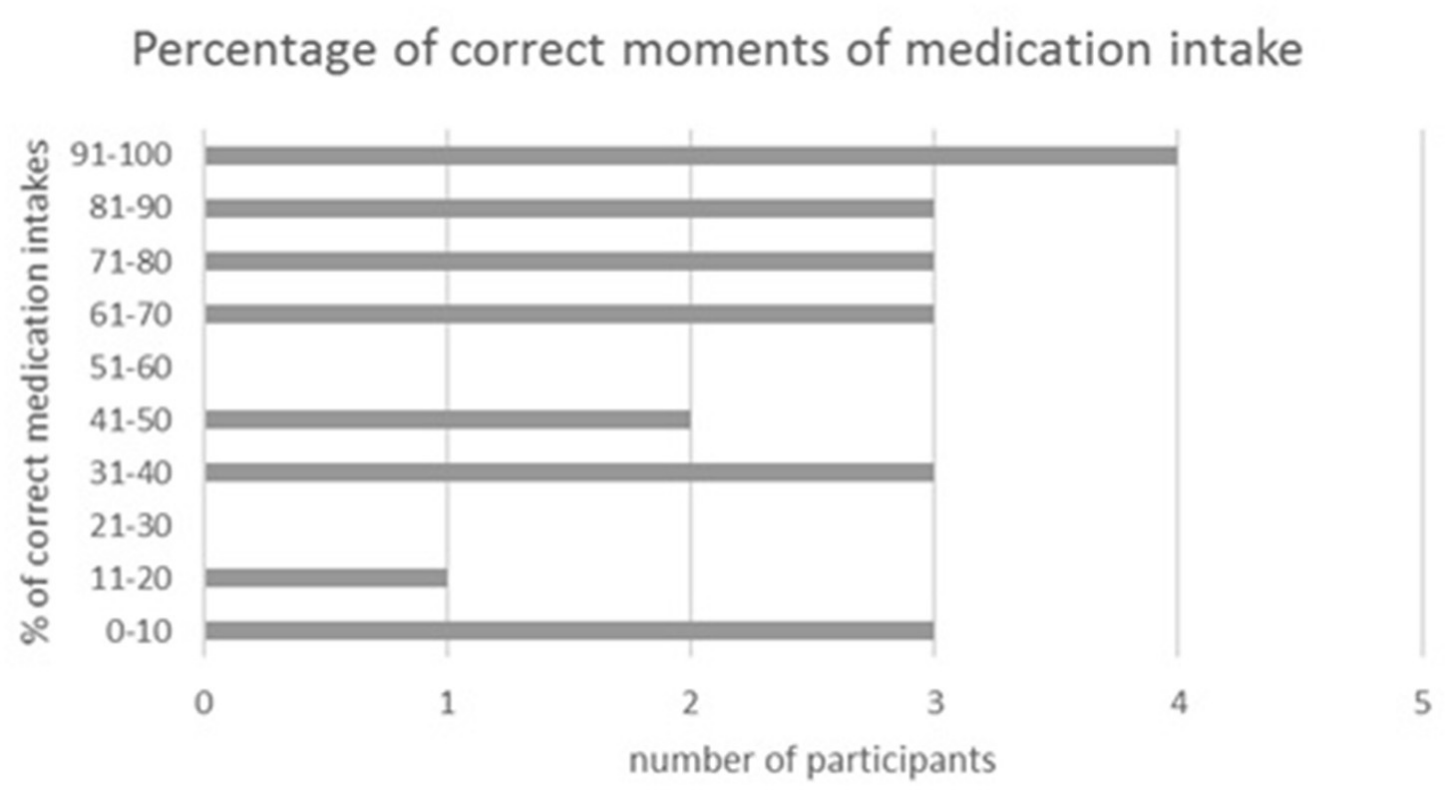
Percentage of correct moments of medication intake. Data are from 2 non-consecutive days, *n* = 22.

Of all food diaries, 22 were eligible for analysis of medication taking behavior. Four patients (18.2%) always took their medication at the right moment, whereas 13.6% never did. The other 15 patients (68.2%) did not take their medication at fixed time points; hence some of the tablets were administered with meals, and others outside meals. Figure 2 provides an overview of the percentage of correct moments of medication intake by the participants.

## Discussion

Nutrient (both macro and micro) intake in PD patients participating in this study was similar to the dietary pattern of the general Belgian population (18). However, our results showed that a large part of the study population had an inadequate intake of vitamin D, thiamin, zinc, and iron. No differences in nutrient intake between men and women was observed, except for vitamin A and copper intake. Regarding the knowledge about food-drug interactions, almost two third of the participants claimed to be aware of the interaction between dietary proteins and levodopa. Nevertheless, <20% took all doses of levodopa outside meals. Our research population however, has a limited sample size, therefore the external validity is limited.

Energy intake of the participants was similar to the average requirements of men and women aged 60–69, based on PAL 1.4–1.8. The energy intake of PD patients in this study was similar to the intake observed in Italian PD patients, although the latter study observed an increase in energy intake with a longer disease duration (19).

Our results showed that carbohydrate intake of PD patients is similar to that of the general Belgian population. In contrast to our results, both Marczewska et al. and Barichella et al. found in Italian PD patients a mean carbohydrate intake of 52.3 and 54.6 E%, respectively (19, 20). This energy contribution of carbohydrates is around 10 E% higher than in our population while for dietary fiber intake the results of Marczewska et al. (20) are similar to ours (26.2 ± 7.7 g vs. 23.8 ± 5.5 g). Barichella et al., however, reported a daily fiber intake of 32.0 ± 8.4 g in PD patients (19). All three studies indicate a remarkable higher fiber intake in PD patients than in healthy controls. Constipation is a common complaint in Parkinson's disease and according to our results more than 50% of the participants changed their diet because of constipation. This might explain the higher intake of dietary fiber in our study population. EFSA states that 25 g dietary fiber per day is an adequate intake to ensure normal laxation in adults (21). However, still 41% of the Parkinson's patients had a daily intake below 25 g.

In PD patients, a positive association was found between an intake of 28 g/day insoluble fiber and levodopa bioavailability (19, 22). Dietary fiber intake impacts on gut health and gut microbiota composition as well (23, 24). The gut microbiome of PD patients differs from healthy age-matched controls, e.g., with regard to butyrate-producing bacteria and butyrate levels (25, 26). A recent study by Matt et al. provided evidence that a high fiber diet reduces neuroinflammation in aging mice (27). The combination of *in vitro*, animal and dietary consumption studies provides preliminary evidence for food-based therapies in the future; a higher dietary fiber intake may relieve some of the symptoms inherent to Parkinson's disease (28).

Our results further demonstrated that the total fat intake in Parkinson's patients is similar to that of the general Belgian population, whereas Barichella et al. found an increased lipid intake, both SFA and PUFA (19). However, Marczewska et al. found no difference in fat intake between PD patients and controls using the European Prospective Investigation into Cancer and Nutrition (EPIC) questionnaire (20).

Due to competition between dietary amino acids and levodopa, Olanow et al. proposed a protein redistribution diet (PRD) in the management of PD (29). In this PRD, protein intake is concentrated in the evening. This improves motor function during the day by reducing levodopa fluctuations. The potential impact of protein intake in the evening is considered less problematic, since mobility is of less importance at night (20, 29). Although PRD can result in a lower protein intake, this diet is considered as safe (30). Our results demonstrated that none of the participants adhered to a low protein diet nor a PRD, since most protein-rich meals were taken at lunch and dinner. In contrast to our results, Marczewska et al. demonstrated a mean daily protein intake of 14.3 E% in PD patients, with an increased level of vegetable protein intake (20). Barichella et al. found an increased protein intake in PD patients compared to controls, which was positively associated with a longer duration of disease (19).

In total, 55.9% of the patients had an inadequate vitamin D intake, which is in line with the low vitamin D intake of the general Belgian population (18). Evatt et al. found an insufficiency in 25-hydroxyvitamin D plasma levels in PD patients compared to healthy controls and Alzheimer's patients (31). Around 10% of the population had an inadequate vitamin B1 intake, which is an issue as studies suggest a relationship between thiamin and dopamine levels. A reduced dopamine release is associated with thiamin deficiency (32, 33). Thiamin deficiency is associated with neuronal loss, and a decrease in the activity of thiamin-dependent enzymes has been demonstrated in neurodegenerative disorders, including PD (34). Although our results clearly show that a vast amount of PD patients has an inadequate intake of several micronutrients, no statements about the extent of the inadequate intake nor of the plasma levels can be made, since potential intake of supplements was not taken into account.

Majority of the patients used levodopa as part of their therapy, which is considered to be the golden standard in PD (7). Although almost 65% of the participants are aware of food-drug interactions between dietary proteins and levodopa, our results clearly show there is room for improvement regarding correct medication intake. A recent study has shown that a pharmacist-led medication review could have a positive impact in this regard (30). When providing patient counseling regarding medication use, a number of topics should be addressed, including the importance of treatment adherence and the appropriate timing of medication intake. A PRD could provide a solution for patients who are not able to take their medication 30 min prior or 1 h after consumption of a meal (e.g., due to nausea), and could reduce motor fluctuations during the day.

The main limitation of this study is the sample size and therefore its restricted external validity. Due to the limited sample size and the inter- and intra-variability of food intake of the participants, the reported nutrient inadequacies should be interpreted with care. Furthermore, patients participating in this study were recruited through their participation in cooking workshops organized by a patient's organization. Therefore, they may have been characterized by a greater interest in food and health, and a higher adherence to a healthier diet, compared to other PD patients. Finally, the dietary records may have been sensitive to misreporting. Nonetheless, this study still provides useful information regarding the dietary intake of Belgian PD patients.

The strength of this study is the use of a dietary record of 2 non-consecutive days, which allows the calculation of usual dietary intake (10, 15, 35). According to our knowledge, this is the first study using a record instead of a recall method.

To conclude, this study, the first in Belgian PD patients, provides evidence that attention for the dietary pattern is key for an optimal dietary management of PD. Our results show that, although the dietary intake of PD patients is similar to that of the general Belgian population, monitoring dietary intake in PD patients is of importance to detect possible micronutrient insufficiencies. Furthermore, we provided evidence that there is room for improvement regarding medication taking behavior in PD patients.

## Data Availability Statement

The datasets presented in this study can be found here: https://doi.org/10.6084/m9.figshare.12646451.

## Ethics Statement

The studies involving human participants were reviewed and approved by 26th of October 2015 by the Ethics Committee of the University of Leuven (Reference MP05560-S58394). The patients/participants provided their written informed consent to participate in this study.

## Author Contributions

CM, RM, DL, and VF developed the study design. FB analyzed obtained data. FB, CM, and GV drafted the manuscript and all authors commented on and approved the final manuscript. All authors are in agreement with the manuscript and declare that the content has not been published elsewhere.

## Conflict of Interest

The authors declare that the research was conducted in the absence of any commercial or financial relationships that could be construed as a potential conflict of interest.

## Footnotes

^1^http://www.parkiskookatelier.be/
